# Патогенные варианты гена <i>TSHR</i> у детей с дисгенезией щитовидной железы

**DOI:** 10.14341/probl13210

**Published:** 2023-02-25

**Authors:** Е. В. Шрёдер, Т. А. Вадина, Е. Н. Солодовникова, В. В. Захарова, М. В. Дегтярев, М. Б. Конюхова, Н. В. Сергеева, О. Б. Безлепкина

**Affiliations:** Национальный медицинский исследовательский центр эндокринологии; Морозовская детская городская клиническая больница; Национальный медицинский исследовательский центр эндокринологии; Национальный медицинский исследовательский центр эндокринологии; Национальный медицинский исследовательский центр эндокринологии; Национальный медицинский исследовательский центр эндокринологии; Морозовская детская городская клиническая больница; Детская поликлиника МБУЗ «Дмитровская городская больница»; Национальный медицинский исследовательский центр эндокринологии

**Keywords:** врожденный гипотиреоз, дисгенезия щитовидной железы, эктопия щитовидной железы, гипоплазия щитовидной железы, рецептор ТТГ, TSHR, резистентность к ТТГ, неаутоиммунный субклинический гипотиреоз

## Abstract

**ОБОСНОВАНИЕ:**

ОБОСНОВАНИЕ. Одна из возможных причин дисгенезии щитовидной железы (ЩЖ) при врожденном гипотиреозе (ВГ) — инактивирующие мутации в гене рецептора тиреотропного гормона (ТТГ) (TSHR) (NP_000360.2). Гетерозиготные мутации гена TSHR приводят к частичной резистентности к ТТГ, гомозиготные и компаунд-гетерозиготные — к ­гипоплазии ЩЖ и полной резистентности. В последние десятилетия в зарубежной литературе появляется все больше данных, касающихся изучения этой проблемы, в то время как отечественные публикации ограничены единичными исследованиями. Изучение этого вопроса необходимо для понимания этиологии, патогенеза заболевания и особенностей тактики наблюдения и лечения пациентов.

**ЦЕЛЬ:**

ЦЕЛЬ. Оценить частоту встречаемости патогенных вариантов гена TSHR у детей с дисгенезией ЩЖ при ВГ, изучить пути наследования заболевания в семьях и оценить фенотипические особенности.

**МАТЕРИАЛЫ И МЕТОДЫ:**

МАТЕРИАЛЫ И МЕТОДЫ. Проведено одноцентровое интервенционное одномоментное несравнительное исследование. Обследованы дети с ВГ, обусловленным дисгенезией ЩЖ. Пациентам проведены УЗИ шеи и радиоизотопная визуализация тиреоидной ткани. Обследование проведено на фоне отмены гормональной терапии либо до ее начала. Произведена оценка структуры дисгенезии ЩЖ, выполнен поиск вариантов в гене TSHR методом NGS, обследованы родители и сибсы пациентов с мутациями гена TSHR.

**РЕЗУЛЬТАТЫ:**

РЕЗУЛЬТАТЫ. В исследование включены 95 детей (75 девочек; 20 мальчиков). Возраст на момент обследования составил 6,2 года [4,5; 8,9], медиана уровня неонатального ТТГ — 157,5 мЕ/л [60,9; 257,2]. Эктопия ЩЖ выявлена у 52% детей, аплазия — у 36%, гипоплазия и гемиагенезия — у 10 и 2% соответственно.

Мутации гена TSHR выявлены в 5,3% случаев (у 5 из 95 детей). Два ребенка имели моноаллельные варианты последовательности (по 1 гетерозиготному варианту — p.R450H и p.D487N), 3 — биаллельные (у 2 пробандов выявлена гомозиготная мутация p.S49Afs*9, 1 ребенок был компаунд-гетерозиготен по p.A485D и p.R450H). У всех пациентов по данным УЗИ имелась гипоплазия ЩЖ разной степени выраженности. Троим детям проведена тиреосцинтиграфия, выявлен сниженный захват радиофармакологического препарата (0,3–0,9%).

Обследование 15 членов семей выявило 9 человек с мутациями гена TSHR, при этом нарушений функции ЩЖ на момент обследования не было выявлено ни в одном случае.

**ЗАКЛЮЧЕНИЕ:**

ЗАКЛЮЧЕНИЕ. Частота мутаций гена TSHR, по нашим данным, составила 5,3%. Выявлены 2 ранее не описанные гетерозиготные мутации. Генетическое исследование может помочь с постановкой диагноза, определением дальнейшего наблюдения и генетического консультирования.

## Abstract (en)

**BACKGROUND:**

BACKGROUND: Loss-of-function mutations in the TSH receptor gene (TSHR) (NP_000360.2) are the potential causes of thyroid dysgenesis in patients with congenital hypothyroidism. Heterozygous variants of the TSHR gene lead to partial resistance to TSH, homozygous and compound heterozygous variants have been shown to cause CH due to thyroid hypoplasia or TSH resistance. Recently more and more articles in this field have appeared in the international literature sources, while local publications are limited. The studies are necessary to understand the etiology, pathogenesis of the disease, to improve the management of these patients.

**AIM:**

AIM: To assess the frequency of incidence of pathogenic variants of the TSHR gene in children with CH due to thyroid dysgenesis. To study inheritance and phenotypic patterns of CH in families.

**MATERIALS AND METHODS:**

MATERIALS AND METHODS: In this single-center interventional one-stage non-comparative study a group of CH patients was examined. The patients underwent neck ultrasound and radionuclide imaging. The examination was performed 14 days after hormone replacement therapy suspension or prior to its initiation. The structure of thyroid dysgenesis was estimated, genetic testing for mutations in the TSHR gene was performed using the NGS method.

**RESULTS:**

RESULTS: The study included 95 children with primary CH (75 girls; 20 boys). The patients’ median age at the time of examination was 6.2 years [4.5; 8.9], the median level of neonatal TSH was 157.5 mU/l [60.9; 257.2]. Ectopic thyroid was found in 52% of children, aplasia in 36%, hypoplasia and hemiagenesis in 10% and 2%, respectively. In 5.4% of cases (in 5 out of 95 patients), different variants of the TSH gene were detected. Two children had heterozygous p.R450H and p.D487N variants in TSHR gene, two patients was homozygous for the p.S49Afs * 9 variant, one child had compound heterozygous variants (p.A485D and p.R450H). According to ultrasound imaging, all patients had thyroid hypoplasia of varying severity. Three children underwent thyroid scintigraphy, which revealed decreased 99mТc pertechnetate uptake (0.3–0.9%).

**CONCLUSION:**

CONCLUSION: In our study, the incidence of different variants in the TSHR gene in children with CH was 5.3%. Our analysis uncovered two previously undescribed variants. Genetic testing may be able to help with making the diagnosis, patient’s management, and genetic counseling.

## ОБОСНОВАНИЕ

Врожденный гипотиреоз (ВГ) у большинства пациентов обусловлен дисгенезией щитовидной железы (ЩЖ) [1–5]. В ее структуре выделяют эктопию, аплазию, гипоплазию и гемиагенезию. Дисгенезия чаще является спорадической, однако в ряде случаев идентифицируется генетическая причина [6].

Одна из возможных причин — мутация в гене рецептора ТТГ (TSHR), который принадлежит к семейству рецепторов, связанных с G-белком (гуанин-нуклеотид-связывающий белок) [6]. В литературе описаны как активирующие, так и инактивирующие мутации в гене TSHR, влияющие на способность ЩЖ синтезировать гормоны. Известно, что инактивирующие мутации вызывают резистентность к тиреотропному гормону (ТТГ) и могут приводить к ВГ [6][7]. В статье приводятся данные пациентов с ВГ, обусловленным инактивирующими мутациями TSHR.

Резистентность к ТТГ представляет собой снижение чувствительности фолликулов ЩЖ к стимуляции ТТГ вследствие генетических дефектов [7]. Гетерозиготные мутации гена TSHR приводят к частичной резистентности к ТТГ, в то время как гомозиготные и компаунд-гетерозиготные — к гипоплазии ЩЖ и полной резистентности к ТТГ [8–12].

В последние десятилетия в зарубежной литературе появляется все больше данных по изучению этой проблемы, проводится анализ частоты и клинических проявлений гипотиреоза. В 2021 г. Da D.-Z. и соавт. [13] опубликован крупный систематический обзор, касающийся изучения патогенных вариантов TSHR при ВГ, частоты их встречаемости и клинических особенностей пациентов с этим генотипом. Отечественные публикации ограничены единичными исследованиями [14–16].

Поскольку ген TSHR является одним из факторов, участвующих в развитии ВГ, обусловленного гипоплазией, его изучение в клинической практике необходимо для понимания этиологии, патогенеза заболевания и особенности тактики ведения пациентов.

## ЦЕЛЬ ИССЛЕДОВАНИЯ

Оценить частоту встречаемости патогенных вариантов гена TSHR у детей с дисгенезией ЩЖ при ВГ, изучить пути наследования заболевания в семьях и оценить фенотипические особенности.

## МАТЕРИАЛЫ И МЕТОДЫ

Место и время проведения исследования

Место проведения. Обследование пациентов проведено в ФГБУ «НМИЦ эндокринологии» Минздрава России (далее — ЭНЦ).

Время исследования. В исследование включены пациенты, обследованные с ноября 2020 г. по июль 2022 г.

Изучаемые популяции

Критерии включения в исследование:

1. дети с ВГ, обусловленным дисгенезией ЩЖ от 0 до 18 лет;

2. родители и сибсы пациентов с мутациями гена TSHR;

3. подписанное информированное согласие на участие в исследовании.

Критерии исключения: отзыв согласия пациента или его законного представителя на участие в исследовании.

Дизайн исследования

Проведено одноцентровое интервенционное одномоментное несравнительное исследование. Набор пациентов в группы проводился на основании соответствия критериям включения и при отсутствии критериев исключения.

На первом этапе определена группа пациентов: в исследование включены дети с ВГ, обусловленным дисгенезией ЩЖ, которым проведены:

1.лабораторное обследование: определение уровней ТТГ, свободного тироксина (св.Т4), тиреоглобулина (ТГ);

2.инструментальная диагностика — комплексная визуализация тиреоидной ткани, сочетающая УЗИ шеи, планарную сцинтиграфию, однофотонную эмиссионную компьютерную томографию (ОФЭКТ) шеи и верхнего средостения с 99mТc-пертехнетатом;

3.молекулярно-генетическое исследование генов-кандидатов, ответственных за развитие ВГ.

Инструментальная диагностика и определение уровня ТГ проводились на фоне отмены гормональной терапии в течение 14 дней.

На втором этапе произведена оценка структуры дисгенезии ЩЖ у детей с ВГ, выполнен поиск вариантов в гене TSHR.

На третьем этапе обследованы родители и сибсы пациентов с выявленными мутациями гена TSHR, которым проведено секвенирование фрагмента ДНК по Сэнгеру при известной мутации. Членам семьи, у которых выявлены мутации этого гена, проведены УЗИ ЩЖ и определение уровней ТТГ и св.Т4.

Описание медицинского вмешательства

Лабораторное обследование — определение уровней ТТГ, св.Т4, ТГ в сыворотке крови проводилось в клинико-диагностической лаборатории ЭНЦ. Лабораторные исследования выполнены на автоматическом иммунохемилюминесцентном анализаторе ARCHITECT i2000sr (Abbott).

УЗИ проводилось врачом ультразвуковой диагностики на аппарате Voluson E8 expert (GE Healthcare) c линейным датчиком 11L. Исследование проведено с использованием цветового и энергетического допплеровского картирования.

Сцинтиграфия ЩЖ (области шеи и верхнего средостения) проводилась в отделении радионуклидной диагностики на гамма-камере ОФЭКТ Discovery NM630 с применением 99mТc-пертехнетата. Доза радиофармпрепарата (РФП) рассчитывалась индивидуально в зависимости от массы тела пациента с помощью калькулятора вводимой активности PedDose в МБк и мКи (https://www.eanm.org/publications/dosage-calculator). Раствор натрия 99mТc-пертехнетата получали путем элюирования генератора 99Мо/99mTc стерильным изотоническим раствором натрия хлорида. Получаемый из генератора РФП вводился внутривенно в процедурном кабинете. Исследование проводилось через 15–20 мин после внутривенного введения РФП на гамма-камере в положении пациента лежа на спине в режиме статического планарного снимка (10 мин) и затем в режиме ОФЭКТ (15 мин). Обработка полученных данных выполнялась на рабочей станции Xeleris (GE Healthcare) c использованием итеративных методов реконструкции данных и получением трехмерного изображения распределения РФП в тканях шеи и верхнего средостения с последующим описанием анатомо-физиологических характеристик визуализирующейся тиреоидной ткани.

Молекулярно-генетическое исследование проводилось в лаборатории генетики моногенных эндокринных заболеваний. Забор крови проводился из локтевой вены вне зависимости от приема пищи в пробирки с консервантом этилендиаминтетраацетатом в концентрации 1,2–2,0 мг на 1 мл крови. Геномную ДНК извлекали роботизированной станцией Allsheng Autopure-96 (Hangzhou Allsheng Instruments Co., Ltd., China) из периферической крови с использованием набора для выделения геномной ДНК из цельной крови NucleoMag Blood (MN). Выделенную ДНК качественно и количественно анализировали с помощью Quant-iT™ dsDNA HS Assay (Invitrogen, Carlsbad, CA, USA) и спектрофотометра Eppendorf Biospectrometer Fluorescence (Eppendorf AG, Germany) соответственно. Подготовку библиотеки ампликонов, подготовку и обогащение матрицы ДНК проводили в соответствии с протоколами производителей (Roche (La Roche Ltd)). Кастомная панель включала в том числе кодирующие области 28 генов: CACNA1C, DUOX1, DUOX2, DUOXA2, FOXE1, GLIS3, GNAS, IGSF1, IYD, KMT2D, NKX2-1, NKX2-5, PAX8, SECISBP2, SLC16A2, SLC26A4, SLC5A5, TBX1, TG, THRA, THRB, TPO, TRH, TRHR, TSHB, TSHR, TTR, UBR1, которые, по научным литературным данным и базе данных из OMIM, были описаны при гипотиреозе. Исследование проведено методом массового параллельного секвенирования (next-generation sequencing, NGS) на платформе Illumina методом парно-концевого чтения (2x150 п.о.).

Обработка данных секвенирования проведена с использованием автоматизированного алгоритма, включающего выравнивание прочтений на референсную последовательность генома человека (HG38), постпроцессинг выравнивания, выявление вариантов и фильтрацию вариантов по качеству, а также аннотацию выявленных вариантов по всем известным транскриптам каждого гена из базы RefSeq с применением компьютерных алгоритмов предсказания патогенности вариантов (SIFT, PolyPhen-2 HDIV, Polyphen-2 HVAR, PROVEAN, CADD). Для оценки популяционных частот выявленных вариантов использованы данные международного проекта gnomAD Exomes для экзонных вариантов и базы gnomAD Genomes для интронных вариантов. Для предсказания эффекта изменений в сайтах сплайсинга и прилежащих к сайту сплайсинга интронных участках использованы программы SpliceAI и AdaBoost.

Для оценки клинической релевантности выявленных вариантов использованы база данных OMIM, HGMD, специализированные базы данных по отдельным заболеваниям (при наличии) и литературные данные. Заключение о клинической значимости найденных вариантов дано с учетом рекомендаций American College of Medical Genetics and Genomics (ACMG) и российского руководства по интерпретации данных NGS. В заключение включены только варианты, имеющие возможное отношение к клиническим проявлениям у пациента. Полиморфизмы, классифицированные по различным критериям как нейтральные, не включены в заключение.

Анализировались панели, средняя глубина покрытия которых была не менее 70x, процент целевых нуклеотидов с эффективным покрытием >10х — не менее 97%.

Стоит отметить, что метод NGS не позволяет достоверно выявлять инсерции и делеции длиной более 10 п.о., мутации в интронных областях (за исключением канонических сайтов сплайсинга), а также мутации в генах, у которых в геноме существует близкий по последовательности паралог (псевдоген). Методика NGS не предназначена для определения фазы пар гетерозиготных мутаций и выявления мутаций в состоянии мозаицизма.

Для подтверждения патогенности обнаруженных вариантов в гене TSHR проведено секвенирование по Сэнгеру ДНК крови родителей и сибсов на генетическом анализаторе Applied Biosystems 3500.

Этическая экспертиза

Исследование одобрено локальным этическим комитетом, протокол №17 от 28.10.2020. Информированное согласие получено от всех обследованных пациентов. В том случае, если возраст обследованных не достиг 15 лет, информированное согласие подписано законным представителем.

## РЕЗУЛЬТАТЫ

В исследование включены 95 детей с ВГ, обусловленным дисгенезией ЩЖ (75 девочек; 20 мальчиков). Возраст на момент обследования составил 6,2 года [ 4,5; 8,9], медиана уровня неонатального ТТГ — 157,5 мЕ/л [ 60,9; 257,2]. Структура дисгенезии (по данным метода комплексной визуализации тиреоидной ткани) представлена на рисунке 1. Эктопия ЩЖ наблюдалась в половине случаев (52%), аплазия ЩЖ имелась у 36% пациентов, гипоплазия и гемиагенезия — у 10 и 2 пациентов соответственно.

По результатам молекулярно-генетического исследования мутации гена TSHR выявлены в 5,3% случаев (у 5 из 95 детей). Два пациента (NN1-2) имели моноаллельные варианты последовательности: пациент 1 имел гетерозиготный вариант p.R450H, а пациент 2 — гетерозиготный p.D487N. Три ребенка (N3-5) имели биаллельные мутации: у 2 пробандов (дети от близкородственного брака — родители троюродные брат и сестра) выявлена гомозиготная мутация p.S49Afs*9, 1 ребенок был компаунд-гетерозиготен по p.A485D и p.R450H. В таблице 1 представлены результаты обследования пациентов.

В неонатальном периоде 3 детей (NN1-3) имели повышение уровня ТТГ по данным скрининга от 10,8 до 15,1 Ед/л, в то время как у ребенка (N5) с гомозиготной мутацией и выраженной гипоплазией ЩЖ выявлено значительное повышение ТТГ — до 96,8 мЕд/л. У всех пациентов по данным УЗИ отмечалась гипоплазия ЩЖ разной степени выраженности. Троим детям проведена тиреосцинтиграфия, по данным которой обращал на себя внимание сниженный захват РФП (со значениями от 0,3 до 0,9%). Суточная доза левотироксина натрия у детей с гетерозиготными и компаунд-гетерозиготной мутациями была ниже, чем у детей с гомозиготной мутацией (табл. 1), что также свидетельствовало в пользу резистентности различной степени выраженности.

Для уточнения цис-/транс-положения найденных вариантов проведены секвенирование по Сэнгеру всем членам семей пробандов (NN1-5) и оценка фенотипа родственников. Обследование членов семьи (n=15) выявило еще 9 человек с мутациями гена TSHR(NP_000360.2): 3 из них были гетерозиготны по p.R450H, 1 — по p.D487N, 1 — по p.A485D, и 4 родителей из 2 семей имели по одной гетерозиготной мутации p.S49Afs*9. Для пациента N3 мы подтвердили компаунд-гетерозиготность мутации. Пациенты N4 и N5 родились от близкородственных браков (родители являются троюродными братом и сестрой). Учитывая обнаружение одинаковой мутации в этих семьях и факт их проживания в одной республике, можно предположить родственную связь этих семей. Родословная пациентов и выявленные изменения представлены на рисунке 2.

У членов семьи, являющихся носителями мутаций, определены уровни ТТГ и св.Т4, нарушений функции ЩЖ не выявлено, объем ЩЖ у родителей варьировал от 6,4 до 18,4 см3. Интересным является то, что у пациента N1 имеется дизиготный близнец 4,2 года, носитель аналогичной мутации, у которого на момент обследования уровни ТТГ и св.Т4 были в пределах нормальных значений, объем ЩЖ — 2,3 см3. Таблица 2 демонстрирует результаты обследования членов семьи.

**Figure:**
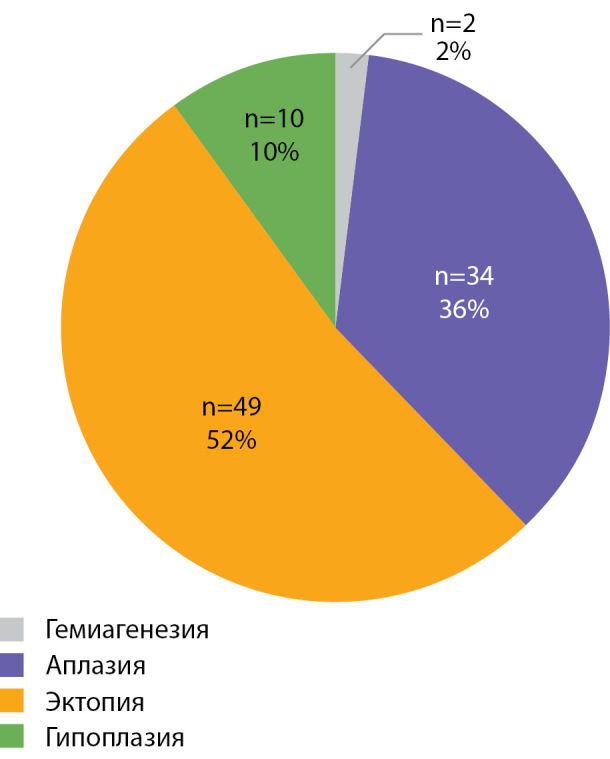
Рисунок 1. Структура дисгенезии щитовидной железы у детей с врожденным гипотиреозом.

**Table:** Таблица 1. Клиническая характеристика пациентов с изменениями в гене TSHR (NM_000369.5)

Пациент	Уровень ТТГ при диагностике ВГ	Результаты обследования в ЭНЦ	Результатымолекулярно-генетического исследования
ТТГ неонатальный, мЕ/л(норма до 10)	ТТГ венозной крови, мЕ/л (подтверждающая диагностика)	Возраст на момент обследования в ЭНЦ, лет	ТГ, нг/мл (норма 3,5–77)	Объем ЩЖ по данным УЗИ, см3	Индекс захвата технеция по данным сцинтиграфии, % (норма 0,8–1,7)	Суточная доза левотироксина натрия, мкг/кг/сут	Изменение аминокислот	Изменение кДНК	Экзон	Зиготность	Классификация ACMG	Описана/ не описана
N1	10,8	14,2	4,2	55,5	1,2	0,3	1,8	R450H	c.1349G>A	10	Het	Патогенный	Описана
N2	12,9	23,4	4,7	30,7	1,6	0,5	2,4	D487N	c.1459G>A	10	Het	Неопределенная клиническая значимость	Не описана
N3	15,1	23,3	4,3	90,1	1,1	0,9	2,5	A485D	c.1454C>A	10	ComHet	Вероятно патогенная	Не описана
R450H	c.1349G>A	10	Вероятно патогенная	Описана
N4	Нет данных	28,4	7,3	0,9	0,1	Не проведена	3,7	S49Afs*9	c.144del	1	Homo	Патогенная	Описана
N5	96,8	317,3	7,0	1,6	0,5	Не проведена	3,5	S49Afs*9	c.144del	1	Homo	Патогенная	Описана

**Figure:**
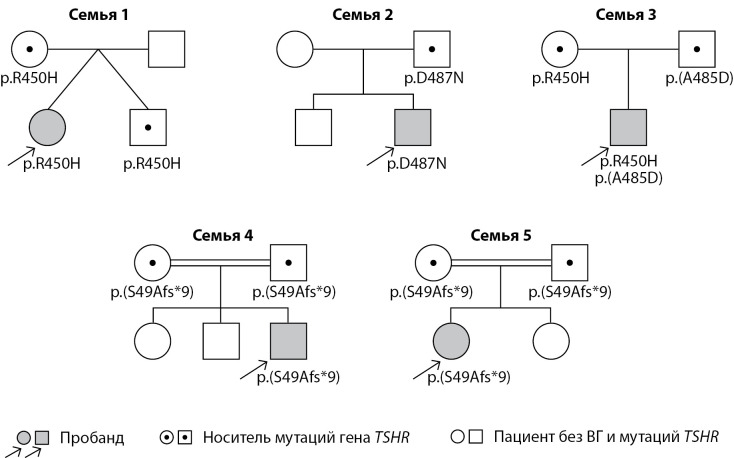
Рисунок 2. Родословные пациентов с мутацией в гене TSHR (NP_000360.2).

**Table:** Таблица 2. Результаты секвенирования по Сэнгеру при известной мутации TSHR (NM_000369.5) и фенотип щитовидной железы

Пациент, N	Возраст на момент обследования, лет	Член семьи	Изменение аминокислот	Изменение кДНК	Экзон	Зиготность	Классификация ACMG	ТТГ, мЕ/л	св.Т4, пмоль/л	Объем ЩЖ, см3
N1	35	Мать	R450H	c.1349G>A	10	Het	Патогенный	2,3	11,4	16,8
52	Отец	Не выявлено	Не выявлено						
4,2	Брат	R450H	c.1349G>A	10	Het	Патогенный	3,1	12,8	2,3
N2	40	Мать	Не выявлено	Не выявлено						
44	Отец	D487N	c.1459G>A	10	Het	Неопределенная клиническая значимость	2,2	12,6	12,3
13	Брат	Не выявлено	Не выявлено						
N3	40	Мать	R450H	c.1349G>A	10	Het	Вероятно патогенная	Не обследована	Не обследована	Не обследована
41	Отец	A485D	c.1454C>A	10	Het	Вероятно патогенная	Не обследован	Не обследован	Не обследован
N4	32	Мать	S49Afs*9	c.144del	1	Het	Патогенный	3,4	14,2	6,4
38	Отец	S49Afs*9	c.144del	1	Het	Патогенный	2,9	14,2	18,4
11	Сестра	Не выявлено	Не выявлено						
10	Брат	Не выявлено	Не выявлено						
N5	29	Мать	S49Afs*9	c.144del	1	Het	Патогенный	2,2	12,9	9,2
34	Отец	S49Afs*9	c.144del	1	Het	Патогенный	1,6	15	11,8
3	Сестра	Не выявлено	Не выявлено						

## ОБСУЖДЕНИЕ

В группе обследованных детей с ВГ, обусловленным дисгенезией ЩЖ, преобладает эктопия — 52%, что согласуется с данными ранее проведенных исследований [3][4][17][18]. Второе место по частоте занимает аплазия (36%). На долю гипоплазии и гемиагенезии приходится 10 и 2% соответственно. Известно, что гипоплазия в ряде случаев обусловлена мутациями в гене TSHR [13].

Согласно систематическому обзору Da D.-Z. и соавт. 2021 г. [13], включавшему анализ данных 3975 детей с ВГ из 23 стран (представленных в 44 исследованиях «случай-контроль»), средняя частота патогенных вариантов в гене TSHR составила 7,83%. Частота патогенных вариантов в гене TSHR зависит от популяций и варьирует от 0 (Бразилия) до 29% (Израиль). Также обращает на себя внимание выраженная вариабельность в рамках одной популяции: в различных исследованиях, проведенных в Италии, она варьирует от 0 до 30,6%. Средняя частота патогенных вариантов в Европе была значительно ниже, чем в Азии.

Частота мутаций гена TSHR у пациентов с гипоплазией ЩЖ в российской популяции составила 5,3%. Наши результаты сопоставимы с результатами, полученными в Японии (Narumi S. и соавт. [19] — 5,88%), в Китае (Fang Y. и соавт. [20] — 5,91%, Ma S.G. и соавт. [21] — 5,56%, Qiu Y.L. и соавт. [22] — 5%) и Корее (Park K.J. и соавт. [23] — 5,29%). Отечественные публикации ограничены, по данным Макрецкой Н.А. и соавт. [15], частота составила 3,73%.

Изучение структуры и функции гена важно для лучшего понимания патогенеза заболевания, а данные о типе наследования и варианте мутации могут помочь в своевременной диагностике нарушений функции ЩЖ у членов семьи.

Известно, что ген TSHR впервые клонирован Parmentier М. и соавт. [24] в 1989 г. и изначально обнаружен у мышей Tshrhyt/hyt как ген, влияющий на дифференцировку ЩЖ [25].

Akamizu Т. и соавт. в 1990 г. [26] картировали ген TSHR на длинном плече 14 хромосомы человека (14q). Rousseau-Merck М. и соавт. [27] и Libert F. и соавт. [28] путем гибридизации in situ локализовали его до 14q31. Ген содержит 10 экзонов, состоит из α- и β-субъединиц, соединенных дисульфидной связью. TSHR представляет собой рецептор, связанный с G-белком, который экспрессируется на базолатеральной мембране тироцитов [29]. Основные его функции — это связывание ТТГ, регуляция роста и пролиферации клеток ЩЖ и участие в синтезе тиреоидных гормонов. ТТГ оказывает свое биологическое действие путем связывания с внеклеточным доменом рецептора ТТГ, расположенного на плазматической мембране тироцитов. Ген TSHR состоит из 7 трансмембранных доменов (transmembrane domain; TMD), соединенных через линкерную область (шарнирную область) с крупным внеклеточным доменом (extracellular domain; ECD), в основном состоящим из последовательности нескольких лейцин-богатых регионов (leucine-rich repeat regions; LRR) (рис. 3). Лейцин-богатые регионы собираются в структуру, похожую на подкову, с бета-нитями LRR, образующими вогнутую поверхность для связывания лиганда. TMD состоит из альфа-спиральных трансмембранных сегментов, соединенных внеклеточными петлями, контактирующими с лигандом ECD, и внутриклеточными петлями, участвующими в связывании G-белка [7]. Крупный ECD кодируется первыми 9 экзонами, а трансмембранные сегменты и карбоксильный конец — 10-м экзоном.

**Figure:**
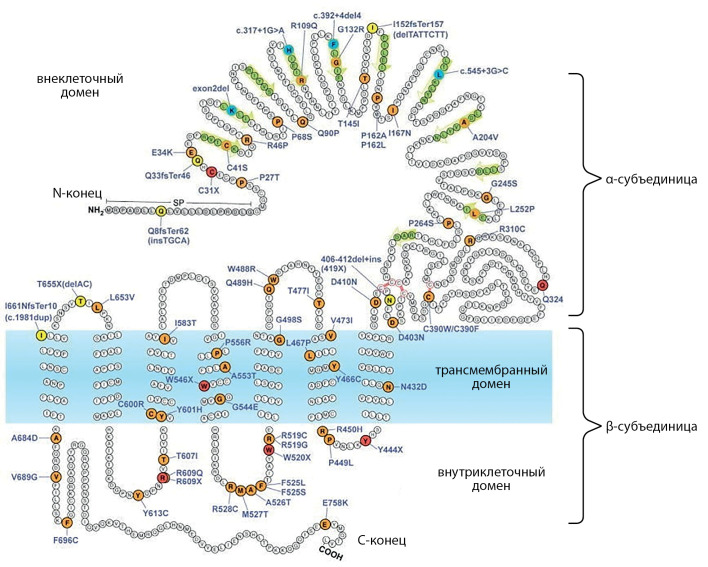
Рисунок 3. Модель TSHR с расположением подтвержденных и предполагаемых инактивирующих мутаций, выявленных у пациентов с резистентностью к ТТГ (адаптирован из [7]; с изменениями).

Инактивирующие мутации могут быть причиной развития ВГ с нарушением роста ЩЖ, приводящего к гипоплазии [7]. Для гипотиреоза, обусловленного мутациями гена TSHR, описаны как аутосомно-доминантный, так и аутосомно-рецессивный типы наследования [6–7].

Резистентность к гену TSHR зависит от зиготности и типа мутации [7][13] и варьирует от субклинического до тяжелого гипотиреоза. Выраженность резистентности к ТТГ связана с типом и локализацией мутации гена TSHR: полная потеря функции вследствие биаллельных инактивирующих мутаций обычно приводит к тяжелому ВГ с характерными клиническими проявлениями, в то время как носители других биаллельных мутаций (гомозиготные или компаунд-гетерозиготы) имеют легкую форму заболевания, проявляющуюся в виде субклинического гипотиреоза [7][30]. Пациенты с моноаллельными дефектами гена TSHR, с учетом частичной компенсации, могут не выявляться при неонатальном скрининге [31]. Таким образом, у пациентов с неаутоиммунным повышением ТТГ и низким или нормальным уровнем св.Т4 при проведении дифференциальной диагностики необходимо помнить о таком редком состоянии, как резистентность к ТТГ. В такой ситуации генетическое исследование может помочь с постановкой диагноза.

У гомозиготных носителей вариантов с потерей функции может наблюдаться выраженная гипоплазия ЩЖ, которая в ряде случаев принимается за аплазию ввиду отсутствия или снижения захвата РФП на сцинтиграфии. В этом случае определяемый уровень сывороточного ТГ позволяет дифференцировать гипоплазию от аплазии ЩЖ [7].

Таким образом, пациенты с гомозиготными вариантами с потерей функции гена TSHR нуждаются в раннем начале заместительной терапии левотироксином в высокой дозе, что демонстрируют пациенты N4 и N5. Пациенты N1–3 демонстрируют более мягкий фенотип и нуждаются в меньшей заместительной дозе препарата.

Все выявленные нами варианты у пробандов 4 и 5 представляют собой мутации с потерей функции (LOF-варианты) [7]. У 2 детей выявлен вариант p.R450H, который наиболее распространен в Азии [32][33]. Мама пациентки N1 по национальности азербайджанка, мама ребенка N3 — киргизка. Изменения у пациентов N4–5 —p.S49Afs*9 описаны лишь в российской популяции [34] и по совокупности данных являются патогенными. Две другие мутации — p.D487N и p.A485D, обнаруженные у пациентов 2 и 3, в литературе ранее не упоминались. Согласно нашим данным, с учетом фенотипа, можно предположить их участие в развитии заболевания, а следовательно, требуется дальнейшее изучение для подтверждения их патогенности.

Секвенирование гена TSHR у членов семей показало, что пациенты с двумя обнаруженными мутациями (гомозиготные и компаунд-гетерозиготные) унаследовали их от здоровых родителей, имеющих одну мутацию в гетерозиготном состоянии и нормальную функцию и объем ЩЖ. В семьях, где у пациента была найдена только одна мутация в гетерозиготном состоянии, родитель — носитель мутации не имел клинических и биохимических признаков гипотиреоза. Учитывая предполагаемый аутосомно-рецессивный тип наследования заболевания, вероятнее всего, второй генетический вариант в таких семьях не был идентифицирован ввиду ограничений метода высокопроизводительного секвенирования и требует дальнейшего поиска другими генетическими методами исследования. Целесообразно динамическое наблюдение членов семьи с гомозиготными мутациями с оценкой функции ЩЖ.

При частичной резистентности к ТТГ в ряде исследований было показано, что наличие повышенного уровня ТТГ может быть достаточным для адекватной выработки гормонов ЩЖ [11][14][35] и не всегда требует назначения заместительной терапии у детей [7].

В 1999 г. создана база данных [36], объединяющая сведения о пациентах с мутациями гена TSHR, включающая клиническую картину и функциональную характеристику мутаций. Последнее обновление базы произведено в 2018 г., зарегистрировано 130 семейных и 60 спорадических случаев нечувствительности к ТТГ с инактивирующими мутациями гена TSHR [37].

## ЗАКЛЮЧЕНИЕ

Частота мутаций гена TSHR у пациентов с ВГ, обусловленным дисгенезией ЩЖ, по нашим данным, составила 5,3% и во всех случаях сопровождалась гипоплазией ЩЖ. Выявлены 2 ранее не описанные мутации — p.D487N и p.A485D и 2 описанные — p.R450H и p.S49Afs*9, участие которых можно предположить в развитии заболевания. Для подтверждения их патогенности необходимы дополнительные функциональные исследования. В работе изучены пути наследования, наиболее вероятен аутосомно-рецессивный тип наследования.

Учитывая полученные данные и результаты других исследований, в случае полной резистентности к ТТГ назначение левотироксина натрия обязательно. В случае мутации гена TSHR с частичной резистентностью к ТТГ (субклинический гипотиреоз) назначение терапии остается дискутабельным, поскольку в большинстве случаев повышенных уровней ТТГ бывает достаточно для поддержания нормальной концентрации тиреоидных гормонов. Терапия калия йодидом в данном случае неэффективна.

Таким образом, определение генетической основы у детей с незначительным повышением ТТГ по данным скрининга и гипоплазией ЩЖ, а также у людей с неаутоиммунным субклиническим гипотиреозом может помочь в постановке диагноза и определить тактику наблюдения.

## ДОПОЛНИТЕЛЬНАЯ ИНФОРМАЦИЯ

Источники финансирования. Исследование выполнено в рамках Государственного задания в части реализации протокола клинической апробации: «Метод гибридной анатомо-функциональной визуализации тиреоидной ткани для топической и функциональной диагностики ВГ у детей» (№2019-15-21), а также при поддержке Благотворительного фонда «Культура благотворительности» в рамках программы «Альфа-Эндо» (молекулярно-генетический блок обследования).

Конфликт интересов. Авторы декларируют отсутствие явных и потенциальных конфликтов интересов, связанных с содержанием настоящей статьи.

Участие авторов. Шредер Е.В. — концепция и дизайн исследования, предоставление материалов исследования и анализ данных, интерпретация результатов, написание статьи; Безлепкина О.Б. — концепция и дизайн исследования, редактирование текста, внесение ценных замечаний; Вадина Т.А. — концепция и дизайн исследования, редактирование текста; Конюхова М.Б., Сергеева Н.В. — предоставление материалов исследования; Дегтярев М.В., Солодовникова Е.В., Захарова В.В. — предоставление материалов исследования, редактирование текста, внесение ценных замечаний.

Все авторы одобрили финальную версию статьи перед публикацией, выразили согласие нести ответственность за все аспекты работы, подразумевающую надлежащее изучение и решение вопросов, связанных с точностью или добросовестностью любой части работы.

Благодарности. Авторы выносят благодарность Благотворительному фонду «Культура благотворительности» в рамках программы «Альфа-Эндо» за финансирование молекулярно-генетического исследования.
